# Addendum: Unravelling cysteine-deficiency-associated rapid weight loss

**DOI:** 10.1038/s41586-025-09282-7

**Published:** 2025-06-26

**Authors:** Alan Varghese, Ivan Gusarov, Begoña Gamallo-Lana, Daria Dolgonos, Yatin Mankan, Ilya Shamovsky, Mydia Phan, Rebecca Jones, Maria Gomez-Jenkins, Eileen White, Rui Wang, Drew R. Jones, Thales Papagiannakopoulos, Michael E. Pacold, Adam C. Mar, Dan R. Littman, Evgeny Nudler

**Affiliations:** 1 Department of Cell Biology, NYU Grossman School of Medicine, New York, NY USA; 2 Department of Biochemistry and Molecular Pharmacology, NYU Grossman School of Medicine, New York, NY USA; 3 Department of Neuroscience and Physiology, Neuroscience Institute, NYU Grossman School of Medicine, New York, NY USA; 4 Division of Advanced Research Technologies, NYU Grossman School of Medicine, New York, NY USA; 5 Rutgers Cancer Institute, Rutgers University, New Brunswick, NJ USA; 6 Department of Molecular Biology and Biochemistry, Rutgers University, Piscataway, NJ USA; 7 Ludwig Princeton Branch, Ludwig Institute for Cancer Research, Princeton University, Princeton, NJ USA; 8 Department of Biology, York University, Toronto, Ontario Canada; 9 Department of Pathology, Laura and Isaac Perlmutter Cancer Center, NYU Langone Health, New York, NY USA; 10 Department of Radiation Oncology and Laura and Isaac Perlmutter Cancer Center, NYU Langone Health, New York, NY USA; 11 Howard Hughes Medical Institute, New York, NY USA

**Keywords:** Fat metabolism, Mitochondria, Metabolomics

Addendum to: *Nature* 10.1038/s41586-025-08996-y Published online 21 May 2025

A study by Lee et al. (Lee, A.H. et al. Cysteine depletion triggers adipose tissue thermogenesis and weight loss. *Nat. Metab.* 10.1038/s42255-025-01297-8 (2025)) similarly reports a 30% weight loss upon cysteine restriction in a Cse knockout mouse model. Like our findings, they observe depletion of CoA and GSH, strong induction of FGF21, comparable changes in adipose tissue and increased lipid utilization.

Furthermore, Lee et al.’s study provides detailed mechanistic insights into adipose tissue changes, including rapid browning, metabolic shifts and signaling pathways driving these processes. This complements our work focused on the biochemical aspects of cysteine depletion by adding valuable details on the physiological responses.

In summary, the combined findings from both studies significantly enhance our understanding of the effects of cysteine depletion and its role in driving rapid weight loss.

A discussion and citation of this study now appear in the article text.

