# Author Correction: Unravelling cysteine-deficiency-associated rapid weight loss

**DOI:** 10.1038/s41586-025-09780-8

**Published:** 2025-12-12

**Authors:** Alan Varghese, Ivan Gusarov, Begoña Gamallo-Lana, Daria Dolgonos, Yatin Mankan, Ilya Shamovsky, Mydia Phan, Rebecca Jones, Maria Gomez-Jenkins, Eileen White, Rui Wang, Drew R. Jones, Thales Papagiannakopoulos, Michael E. Pacold, Adam C. Mar, Dan R. Littman, Evgeny Nudler

**Affiliations:** 1 Department of Cell Biology, NYU Grossman School of Medicine, New York, NY USA; 2 Department of Biochemistry and Molecular Pharmacology, NYU Grossman School of Medicine, New York, NY USA; 3 Department of Neuroscience and Physiology, Neuroscience Institute, NYU Grossman School of Medicine, New York, NY USA; 4 Division of Advanced Research Technologies, NYU Grossman School of Medicine, New York, NY USA; 5 Rutgers Cancer Institute, Rutgers University, New Brunswick, NJ USA; 6 Department of Molecular Biology and Biochemistry, Rutgers University, Piscataway, NJ USA; 7 Ludwig Princeton Branch, Ludwig Institute for Cancer Research, Princeton University, Princeton, NJ USA; 8 Department of Biology, York University, Toronto, Ontario Canada; 9 Department of Pathology, Laura and Isaac Perlmutter Cancer Center, NYU Langone Health, New York, NY USA; 10 Department of Radiation Oncology and Laura and Isaac Perlmutter Cancer Center, NYU Langone Health, New York, NY USA; 11 Howard Hughes Medical Institute, New York, NY USA

**Keywords:** Fat metabolism, Mitochondria, Metabolomics

Correction to: *Nature* 10.1038/s41586-025-08996-y Published online 21 May 2025

In the version of the article initially published, there was a copy-paste error in Extended Data Fig. 2f,g. Specifically, the muscle H&E panel for Day 3 Het No Cys was identical to the Day 7 KO Ctrl diet, and the Day 3 KO Ctrl panel was identical to the Day 7 KO No Cys. These errors involved the incorrect panels for Day 3 Het No Cys and Day 3 KO Ctrl. The figure has now been updated with the correct panels in both the HTML and PDF versions of the article. This error did not affect the conclusions of the figure or the overall paper.

