# Supplementary material for: Unravelling cysteine-deficiency-associated rapid weight loss
Source: Nature. 2025 May 21;643(8072):776–84. doi: 10.1038/s41586-025-08996-y (PMC12267064; doi:10.1038/s41586-025-08996-y)
Supplement: Supplementary file 1 — Raw gel images for all western blots and replicates. [file 41586_2025_8996_MOESM1_ESM.pdf]

nature portfolio

https://doi.org/10.1038/s41586-025-08996-y

Supplementary information

Unravelling cysteine-deficiency-associated rapid weight loss

In the format provided by the authors and unedited

eIF2α and P-eIF2α Repeat 1:Raw Gel

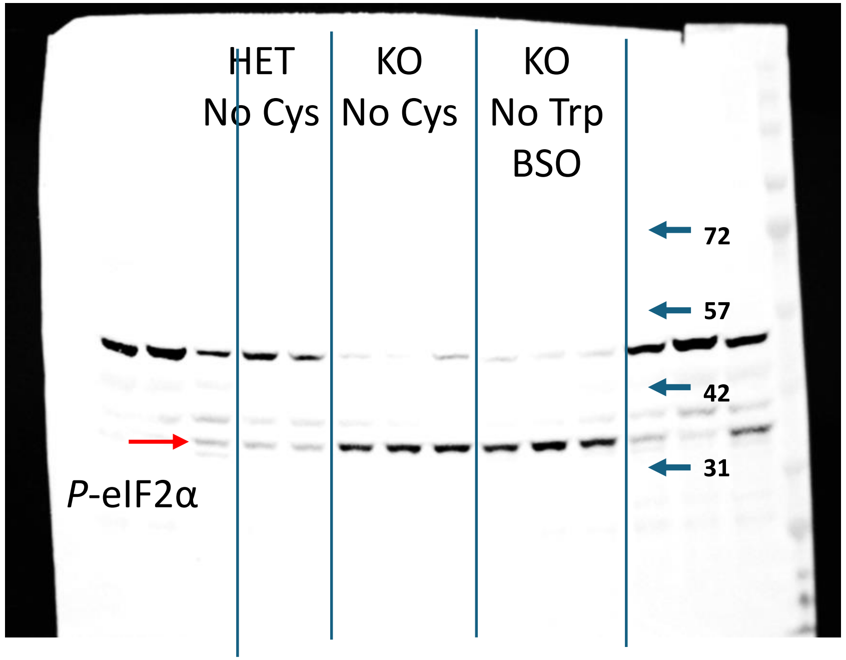

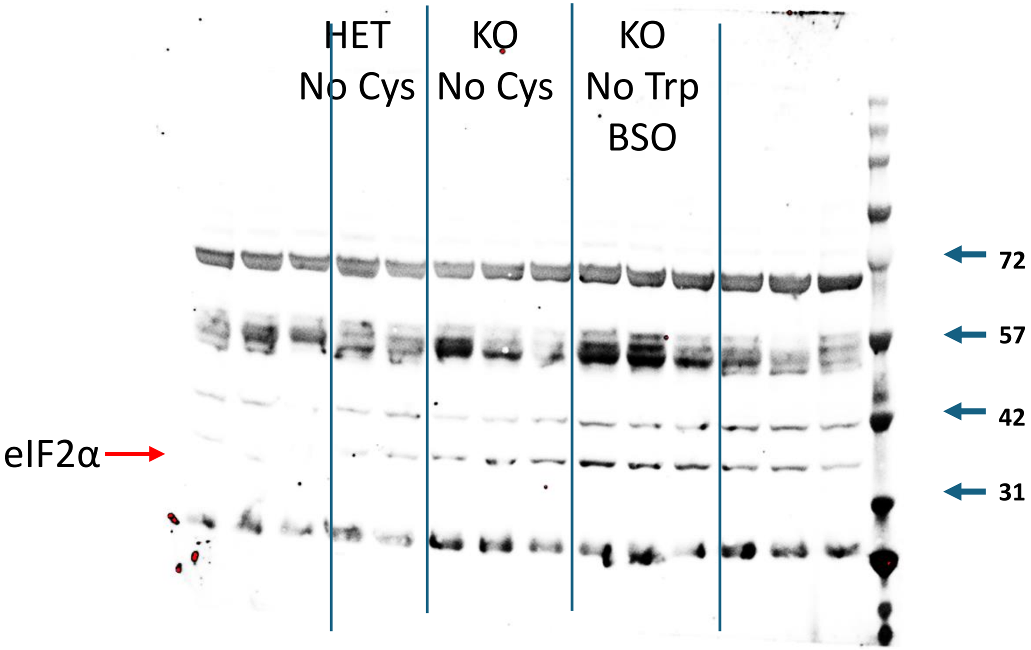

eIF2α and P-eIF2α Repeat 1

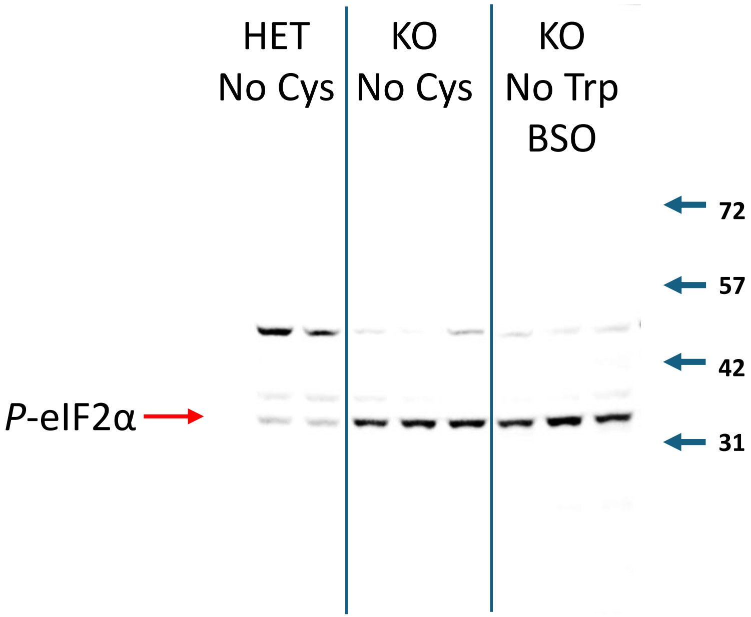

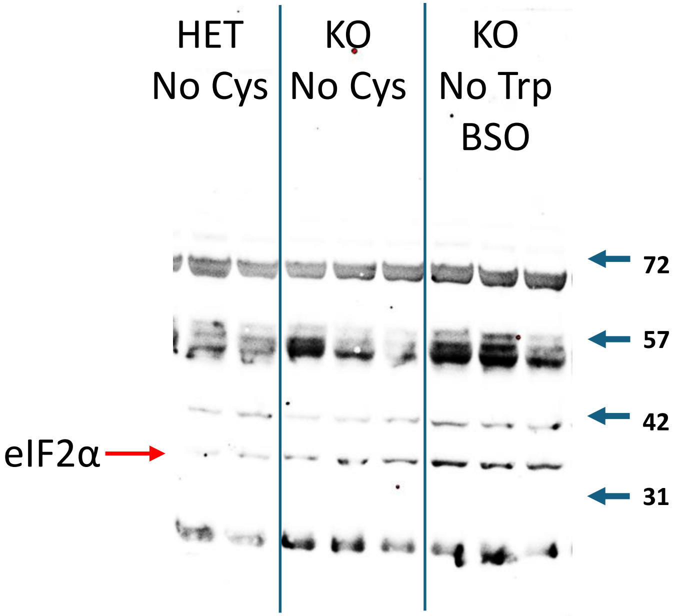

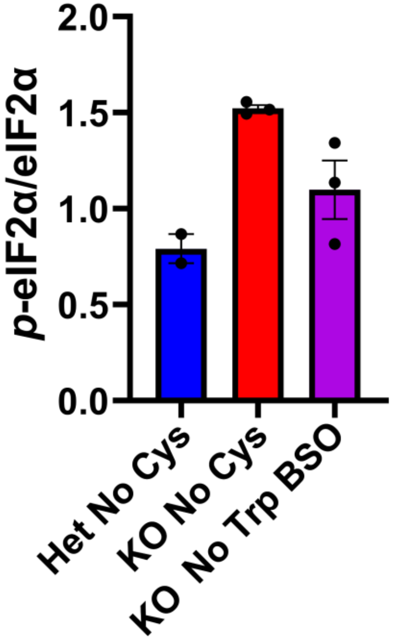

eIF2α and P-eIF2α Repeat 2:Raw Gel

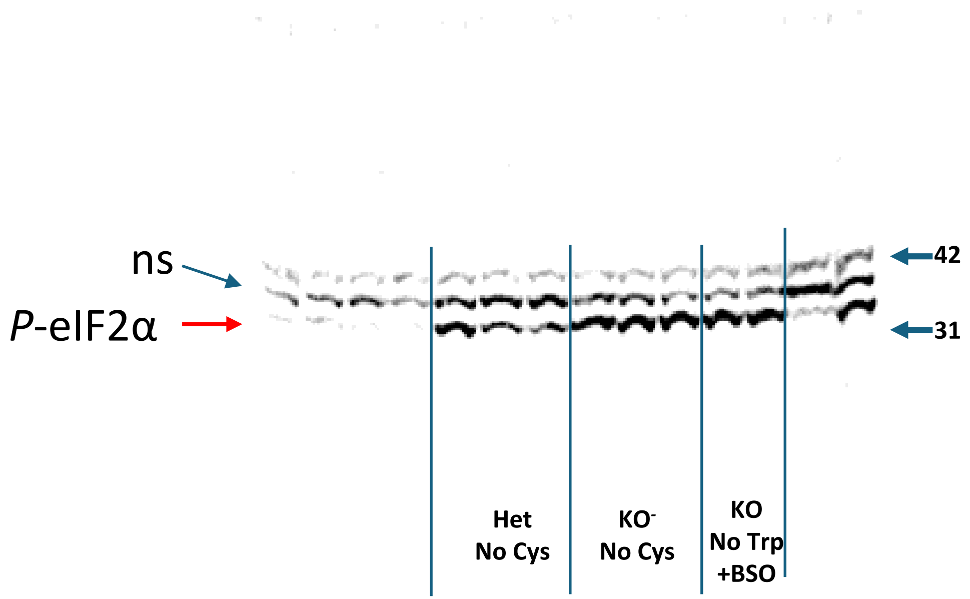

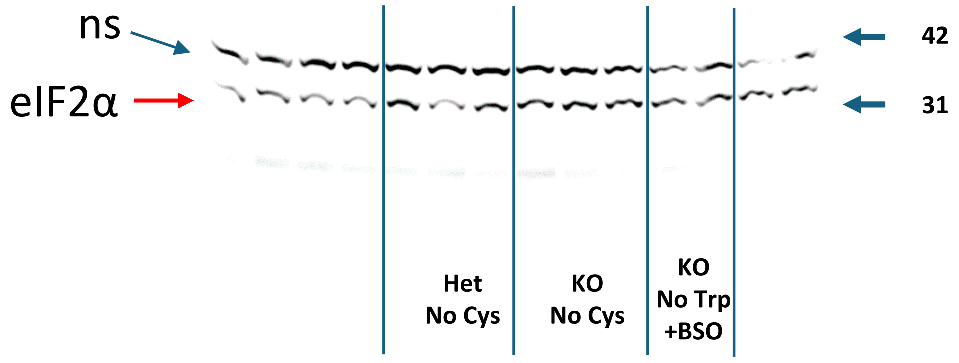

eIF2α and P-eIF2α: Repeat 2

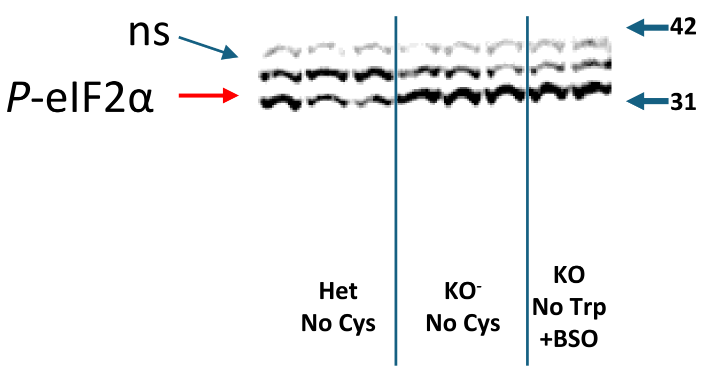

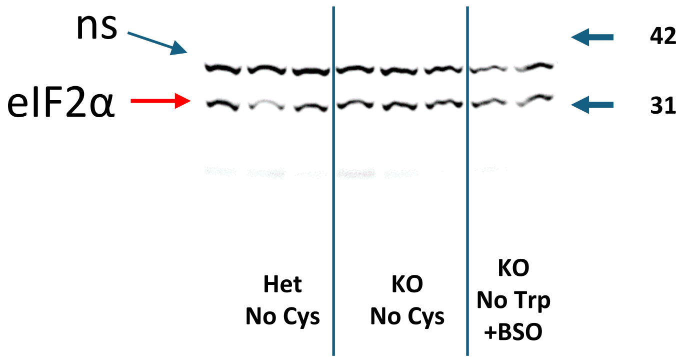

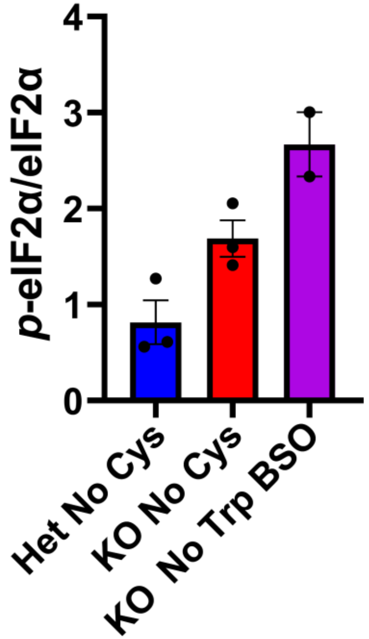

ACC Run-1:Raw gel

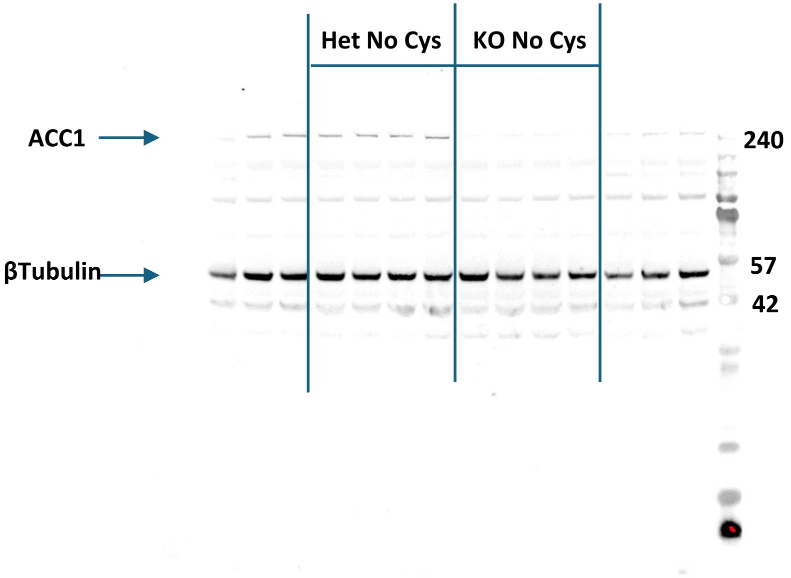

ACC Run-1

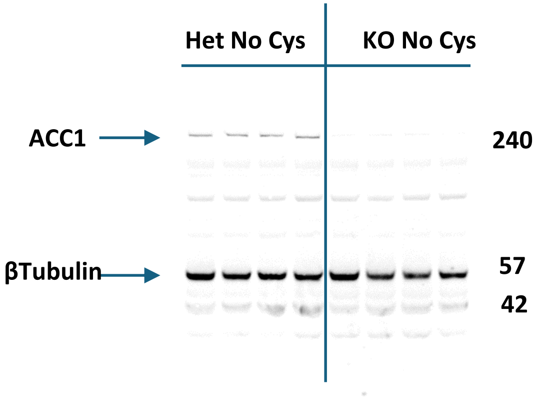
Het No Cys
KO No Cys
ACC1
240
βTubulin
57
42

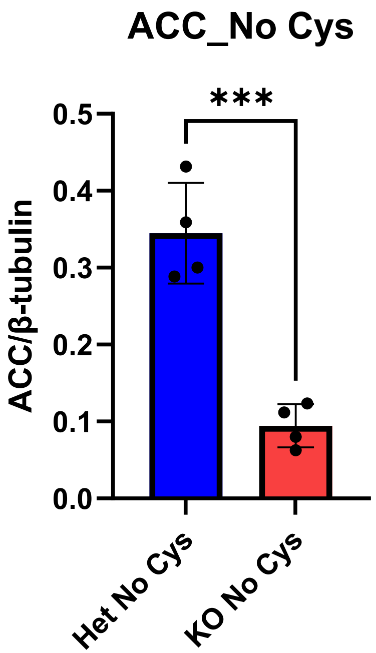
ACC_No Cys
***
ACC/β-tubulin
0.5
0.4
0.3
0.2
0.1
0.0
Het No Cys
KO No Cys

ACC Run-2: Raw Gel

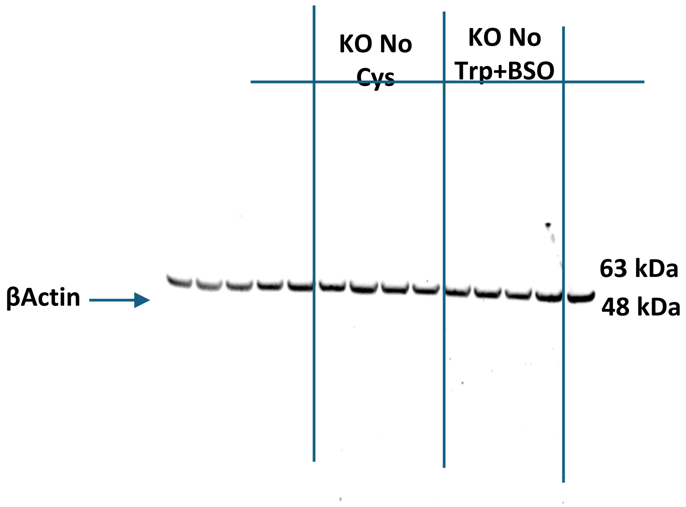

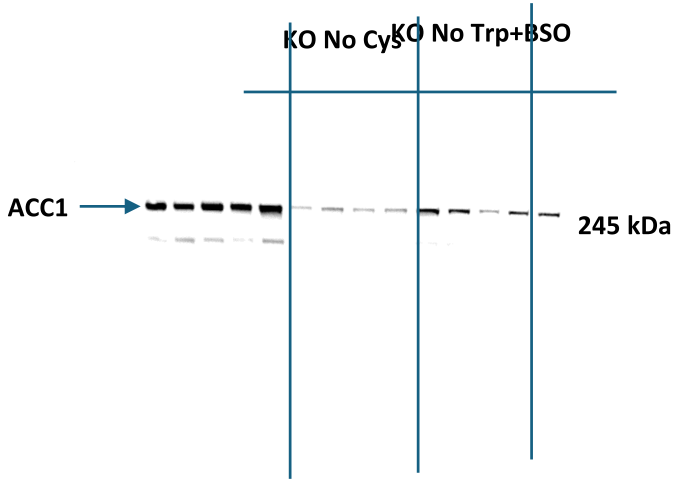

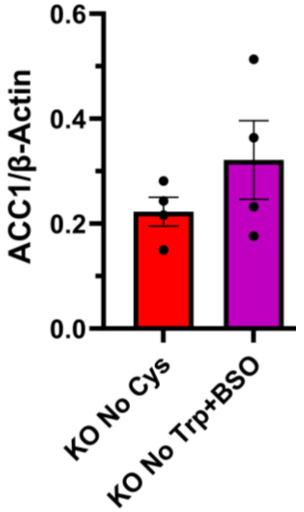

ACC Run-2

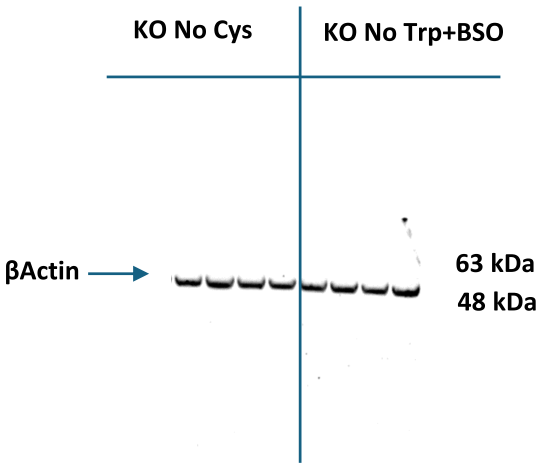
KO No Cys
KO No Trp+BSO
βActin
63 kDa
48 kDa

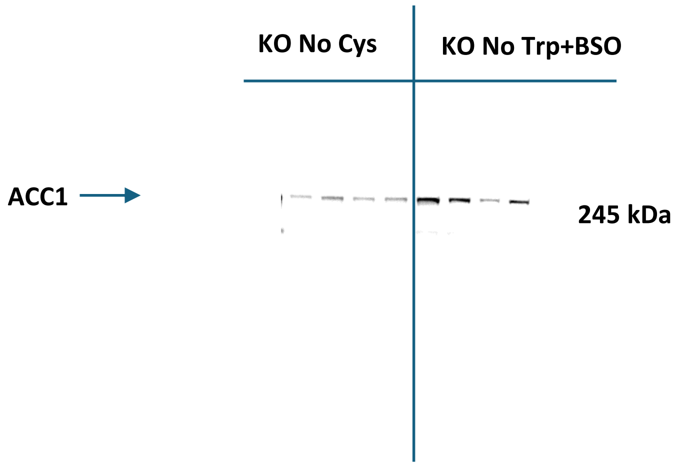
KO No Cys
KO No Trp+BSO
ACC1
245 kDa

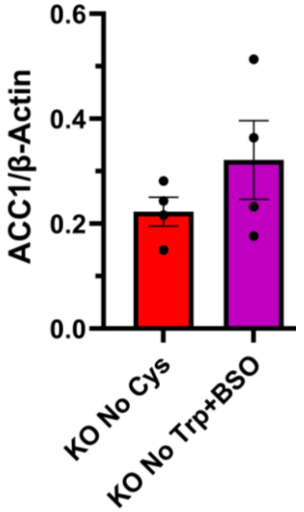
0.6
0.4
0.2
0.0
ACC1/β-Actin
KO No Cys
KO No Trp+BSO
