# Supplementary material for: Unravelling cysteine-deficiency-associated rapid weight loss
Source: Nature. 2025 May 21;643(8072):776–84. doi: 10.1038/s41586-025-08996-y (PMC12267064; doi:10.1038/s41586-025-08996-y)
Supplement: Supplementary file 2 — Reporting Summary [file 41586_2025_8996_MOESM2_ESM.pdf]

nature portfolio

Corresponding author(s): Dan Littman
Evgeny Nudler

Last updated by author(s): 2025/03/07

Reporting Summary

Nature Portfolio wishes to improve the reproducibility of the work that we publish. This form provides structure for consistency and transparency in reporting. For further information on Nature Portfolio policies, see our Editorial Policies and the Editorial Policy Checklist.

Statistics

For all statistical analyses, confirm that the following items are present in the figure legend, table legend, main text, or Methods section.

n/a	Confirmed	
☐	☒	The exact sample size (n) for each experimental group/condition, given as a discrete number and unit of measurement
☐	☒	A statement on whether measurements were taken from distinct samples or whether the same sample was measured repeatedly
☐	☒	The statistical test(s) used AND whether they are one- or two-sided<br>*Only common tests should be described solely by name; describe more complex techniques in the Methods section.*
☐	☒	A description of all covariates tested
☐	☒	A description of any assumptions or corrections, such as tests of normality and adjustment for multiple comparisons
☐	☒	A full description of the statistical parameters including central tendency (e.g. means) or other basic estimates (e.g. regression coefficient) AND variation (e.g. standard deviation) or associated estimates of uncertainty (e.g. confidence intervals)
☐	☒	For null hypothesis testing, the test statistic (e.g. F, t, r) with confidence intervals, effect sizes, degrees of freedom and P value noted<br>*Give P values as exact values whenever suitable.*
☒	☐	For Bayesian analysis, information on the choice of priors and Markov chain Monte Carlo settings
☒	☐	For hierarchical and complex designs, identification of the appropriate level for tests and full reporting of outcomes
☐	☒	Estimates of effect sizes (e.g. Cohen's d, Pearson's r), indicating how they were calculated

Our web collection on statistics for biologists contains articles on many of the points above.

Software and code

Policy information about availability of computer code

Data collection	DEXA Insight, Chemi Doc
Data analysis	Prism (GraphPad, v10.0.2), ImageJ

For manuscripts utilizing custom algorithms or software that are central to the research but not yet described in published literature, software must be made available to editors and reviewers. We strongly encourage code deposition in a community repository (e.g. GitHub). See the Nature Portfolio guidelines for submitting code & software for further information.

Data

Policy information about availability of data

All manuscripts must include a data availability statement. This statement should provide the following information, where applicable:

- Accession codes, unique identifiers, or web links for publicly available datasets
- A description of any restrictions on data availability
- For clinical datasets or third party data, please ensure that the statement adheres to our policy

Source data will be provided with this paper. Accession codes for sequencing data and metabolomics data are provided. All other data supporting the findings of this study are available from the corresponding authors on reasonable request.

Research involving human participants, their data, or biological material

Policy information about studies with human participants or human data. See also policy information about sex, gender (identity/presentation), and sexual orientation and race, ethnicity and racism.

Reporting on sex and gender	N/A
Reporting on race, ethnicity, or other socially relevant groupings	N/A
Population characteristics	N/A
Recruitment	N/A
Ethics oversight	N/A

Note that full information on the approval of the study protocol must also be provided in the manuscript.

Field-specific reporting

Please select the one below that is the best fit for your research. If you are not sure, read the appropriate sections before making your selection.

☒ Life sciences ☐ Behavioural & social sciences ☐ Ecological, evolutionary & environmental sciences

For a reference copy of the document with all sections, see nature.com/documents/nr-reporting-summary-flat.pdf

Life sciences study design

All studies must disclose on these points even when the disclosure is negative.

Sample size	Three or more mice per group were used in each experiment. The precise number of animals used are given in the figure legend. The sample size was determined from our previous experience and from what is accepted in the field. Power analysis was done to confirm numbers used were sufficient.
Data exclusions	No samples were excluded from the analysis.
Replication	Experiments were replicated at least two times.
Randomization	Allocation into sample groups was random. In addition, experimental and control mice were used from the same litter whenever possible. Both males and females were used.
Blinding	A.V. performed majority of mouse experiments and gave samples to I.G. in a blinded manner for metabolite extraction and analysis.

Reporting for specific materials, systems and methods

We require information from authors about some types of materials, experimental systems and methods used in many studies. Here, indicate whether each material, system or method listed is relevant to your study. If you are not sure if a list item applies to your research, read the appropriate section before selecting a response.

Materials & experimental systems

n/a	Involved in the study	
☐	☒	Antibodies
☒	☐	Eukaryotic cell lines
☒	☐	Palaeontology and archaeology
☐	☒	Animals and other organisms
☒	☐	Clinical data
☒	☐	Dual use research of concern
☒	☐	Plants

Methods

n/a	Involved in the study	
☒	☐	ChIP-seq
☒	☐	Flow cytometry
☒	☐	MRI-based neuroimaging

Antibodies

Antibodies used	anti-Phospho-eIF2α (Cell Signaling Technology 3597), anti-eIF2α (Cell Signaling Technology 2103) anti-Phospho-ACC (Cell Signaling Technology 3661), anti-ACC (Cell Signaling Technology 3662), anti-βTubulin (Proteintech 10068-1-AP), Anti-β-Actin–Peroxidase

	antibody (Sigma A3854), UCP1 (CST, Cat#: 72298S, Clone: E9Z2V), CASP3 (CST, Cat#: 9579S, Clone: D3E9), NQO1 (Sigma-Aldrich, HPA007308), Custom NRF2 (1:1000, provided by Edward Schmidt, Montana State University)
Validation	All Antibodies except anti-NRF2 were used according to recommendations of the manufacturer. Anti-NRF2 antibody usage was optimized using Keap1 mutant (Nrf2 overproducing) and Nrf2 KO tumor cell lines.

Animals and other research organisms

Policy information about studies involving animals; ARRIVE guidelines recommended for reporting animal research, and Sex and Gender in Research

Laboratory animals	Mice were bred and maintained in the Alexandria Center for the Life Sciences animal facility of the New York University School of Medicine, in specific pathogen-free conditions. C57BL/6 mice (Jax 000664), Gcn2 KO (B6.129S6-Eif2ak4tm1.2Dron/J) and Fgf21 KO (B6.129Sv(Cg)-Fgf21tm1.1Djm/J) were purchased from Jackson Laboratories. Cse KO (129/C57BL/6 background) were generated by Rui Wang as previously described and given to us by Christopher Hine.Gdf15 KO mice were generated by Eileen White's lab and is described in the methods section. Mice in all the experiments were at least 9 weeks old at the starting point of various diets unless described specifically.
Wild animals	N/A
Reporting on sex	Both males and females were used for experiments. Most weight curves shown are exclusively from male mice or female mice as there is a small variation in weight loss on day 1. To reduce variation during RNA-seq and metabolomics, only male mice were used.
Field-collected samples	N/A
Ethics oversight	All animal procedures were performed in accordance with protocols approved by the Institutional Animal Care and Usage Committee of New York University School of Medicine.

Note that full information on the approval of the study protocol must also be provided in the manuscript.

Plants

Seed stocks	N/A
Novel plant genotypes	N/A
Authentication	N/A
